# Discrete Bayesian Inference as a Structure of Paths

**DOI:** 10.3390/e28050553

**Published:** 2026-05-14

**Authors:** Valerian V. Popkov

**Affiliations:** Independent Researcher, 16900 Prague, Czech Republic; presidentibi829@gmail.com

**Keywords:** Bayesian inference, discrete posterior, combinatorial probability, finite resolution, divergence, representational regimes, prior strength, mesoscopic regime

## Abstract

Bayesian inference is predominantly formulated in a continuous framework, in which posterior beliefs are represented by smooth probability densities. However, an alternative discrete representation—already implicit in Bayes’s original construction—remains conceptually distinct and structurally informative. This paper develops a representation-level analysis of Bayesian updating in the binomial setting and shows that discrete and continuous posteriors may exhibit qualitatively distinct behavior under finite parameter resolution. In particular, coarse discretization can induce regime-dependent divergence from the continuous posterior, even when the algebraic form of the likelihood is identical. The analysis further demonstrates that divergence is not determined solely by grid resolution but also by the balance between prior strength and sample size. By introducing a scale-dependent perspective in which representational resolution and prior magnitude jointly define distinct regimes of inference, the paper clarifies how structural and analytic descriptions interact under finite conditions.

## 1. Introduction

Bayesian inference is predominantly formulated in a continuous framework, in which posterior beliefs are represented by smooth probability densities and updated through analytic integration [1,2]. In the classical binomial setting, a uniform prior on the interval [0,1] yields a Beta posterior density, and this Laplacian representation has become the canonical form in both theoretical and applied Bayesian analysis [3]. Continuous posteriors provide a compact analytic description, enable asymptotic arguments, and support a wide range of computational techniques designed for parametric models.

Historically, however, Bayesian inference did not originate in an analytic continuum. In Bayes’s original essay [4], posterior support was constructed over a finite set of admissible hypotheses, with probabilities derived from the enumeration of possible outcome sequences. Price’s subsequent discussion of Bayes’s rule [5] and Bellhouse’s historical account [6] further contextualize the early development of this construction. The passage from this finite representation to Laplace’s analytic formulation marked a shift not only in mathematical technique but in representational structure. Discrete hypothesis spaces were replaced by infinitely divisible parameter domains, and combinatorial aggregation was replaced by integration. Later accounts and foundational discussions of Bayesian statistics provide additional background for this transition [7,8].

In contemporary Bayesian practice, the continuous regime dominates both theory and computation. Even when discrete approximations are employed—such as grid-based methods or finite particle systems—they are often regarded as numerical surrogates for an underlying continuous model. Convergence arguments justify this view in the limit of infinite refinement. Yet in any concrete inferential setting, both data and representational resolution are finite. Parameters are discretized, rounded, or sampled; posterior summaries are computed from finite representations; and prior influence interacts with limited information.

This observation motivates a distinction between approximation and representation. In the present context, “representation” refers to how posterior support is encoded under finite conditions, whereas “approximation” refers to numerical proximity to an underlying continuous description. Terms such as “admissible hypotheses” and “mesoscopic regime” are used to describe structural features of posterior behavior that arise when parameter resolution and posterior concentration interact. Two posterior constructions may converge numerically as resolution increases, yet differ structurally in how uncertainty is encoded. In the discrete regime, posterior support is organized over admissible hypotheses grounded in combinatorial multiplicities. In the continuous regime, uncertainty is encoded through analytic curvature of a smooth density. These are not merely different computational implementations of the same object; they are distinct representational modes.

The present paper develops a representation-level analysis of Bayesian updating in the binomial setting. The main contribution of this work is not a modification of Bayesian updating itself, but the identification of distinct representational regimes arising under finite conditions. These regimes are determined by the interaction between parameter resolution, sample size, and prior strength, and lead to qualitative differences between discrete and continuous posterior behavior even when the underlying likelihood is identical. In this context, the analysis is intentionally developed within the classical binomial setting, which serves as a minimal framework for isolating representational effects without additional model complexity. This distinction is also relevant in practical contexts where posterior quantities are obtained from finite representations, such as discretized parameter spaces or computational approximations, and may therefore reflect not only numerical but also structural differences. The central thesis is that Bayesian inference admits distinct structural regimes governed by interacting scales: representational resolution, sample size, and prior strength. When resolution is coarse relative to posterior concentration, structural constraints dominate; when resolution is sufficiently fine, analytic smoothing prevails; and between these extremes lies an intermediate mesoscopic regime in which structural and analytic components interact.

This mesoscopic regime is not visible in purely asymptotic treatments. Convergence theorems ensure that discrete posteriors approximate continuous densities as resolution increases for fixed sample size. However, when both sample size and representational resolution vary, different scaling relations produce qualitatively distinct behaviors. Divergence between discrete and continuous posteriors may arise even though the likelihood and updating rule are identical.

A further structural dimension is introduced by prior strength. In the binomial model with a Beta prior, the parameters α and β can be interpreted as effective pseudo-counts [2]. The balance between prior magnitude and sample size determines whether inference is data-dominated, prior-dominated, or intermediate. When combined with representational resolution, prior strength defines a two-dimensional scale structure governing Bayesian inference under finite conditions.

By articulating this regime structure explicitly in a simple classical setting, the paper clarifies how structural and analytic descriptions interact and how finite-resolution effects may influence posterior behavior. Although the binomial model is elementary, it provides a transparent environment in which representational distinctions can be examined without additional modeling complexity. The insights obtained extend beyond this specific model and illuminate how finite-scale considerations may shape Bayesian inference more generally.

The paper is organized as follows. Section 2 formulates the discrete posterior as a structural representation induced by admissible hypotheses and admissible histories. Section 3 reviews the continuous posterior density as an analytic representation and clarifies how analytic smoothing suppresses discrete structure. Section 4 develops numerical regimes under finite resolution. Section 5 provides a scale-based account of divergence and introduces the mesoscopic regime through comparison of posterior width and grid spacing, including scaling pathways. Section 6 analyzes the scale-dependent role of the prior and shows how prior strength interacts with representational resolution in determining inference regimes. The Section 7 summarizes the regime picture and its implications for finite-resolution Bayesian inference.

## 2. Discrete Posterior as Structural Representation

In the discrete formulation of Bayesian inference, the parameter space is represented by a finite set of admissible hypotheses. In the binomial setting, this may be formalized by introducing a grid

(1) $$p_{i}=\frac{i}{N},\;i=0,1,\ldots ,N,$$

which defines a finite collection of possible values for the probability of success. In this analysis, the parameter p is used directly in order to maintain a canonical representation corresponding to the classical binomial model, without introducing additional effects related to reparameterization. In the present paper, equally spaced admissible hypotheses are used as a transparent illustrative choice that makes the representational scale explicit through the grid spacing. This choice is not intended as a general prescription, but as a simple construction that allows the effect of finite resolution to be clearly examined. Each admissible hypothesis corresponds to a distinct structural configuration within the space of possible outcome sequences.

Consider a sequence of *n* Bernoulli trials with *k* observed successes. For a fixed hypothesis $p_{i}$, the likelihood of observing exactly *k* successes is given by

(2) nkpik(1−pi)n−k.

This expression has two conceptually distinct components. The factor $p_{i}^{k}{\left(1-p_{i}\right)}^{n-k}$ reflects the probability assigned by the hypothesis $p_{i}$ to a specific ordered sequence containing *k* successes and n−k failures. The binomial coefficient nk counts the number of distinct admissible sequences that share this success–failure composition.

Thus, the posterior weight assigned to a hypothesis does not arise from a single probabilistic evaluation, but from an aggregation over all admissible histories consistent with the observed data. The discrete posterior can therefore be written as

(3) πN(pi∣k,n)=nkpik(1−pi)n−k∑j=0Nnkpjk(1−pj)n−k.

Since the combinatorial factor is common to all hypotheses, it cancels under normalization, yielding

(4) $$\pi_{N}\left(p_{i}|k,n\right)=\frac{p_{i}^{k}{\left(1-p_{i}\right)}^{n-k}}{\sum_{j=0}^{N}p_{j}^{k}{\left(1-p_{j}\right)}^{n-k}}.$$

Although this cancellation makes the final algebraic form appear identical to the continuous expression evaluated on grid points, the structural interpretation remains different. The binomial coefficient is not a redundant artifact; it expresses the multiplicity of admissible histories. Its cancellation in normalization does not eliminate its conceptual role in the formation of posterior support.

In this sense, the discrete posterior encodes two layers of information: the probabilistic evaluation of individual sequences under each hypothesis and the combinatorial organization of admissible histories. The resulting posterior distribution therefore reflects not only the curvature of a likelihood function, but also the geometry of the discrete hypothesis space.

This structural perspective becomes particularly transparent when the parameter grid is coarse. In such cases, each admissible hypothesis represents a relatively large region of the parameter space, and posterior mass reflects how many admissible histories are consistent with that region. The posterior is therefore shaped by both likelihood and admissibility constraints imposed by finite resolution.

It is important to emphasize that the discrete formulation is not merely a numerical approximation of a continuous density. It constitutes a coherent representational regime in which hypotheses are finite and admissibility plays an explicit role. When resolution is finite, the posterior support is organized over a discrete structural skeleton, and inference proceeds through normalization across this finite set of admissible configurations.

This interpretation provides the foundation for the subsequent analysis. The question is not whether discrete posteriors converge to continuous densities under refinement—they do—but how representational structure influences posterior behavior when resolution is finite. The next section turns to the continuous regime in order to clarify how analytic smoothing suppresses explicit structural distinctions present in the discrete formulation.

*Illustrative Case: A Minimal Combinatorial Structure (n=3)*

To make the structural character of the discrete posterior explicit, consider a minimal example with n=3 Bernoulli trials. Suppose that k=2 successes are observed. There are exactly three admissible ordered outcome sequences consistent with this observation:

SSF,SFS,FSS.

For a fixed hypothesis $p_{i}$, each of these sequences has probability $p_{i}^{2}\left(1-p_{i}\right)$. The total likelihood weight assigned to hypothesis $p_{i}$ is therefore $3\;p_{i}^{2}\left(1-p_{i}\right)$, where the factor 3 arises from the number of admissible histories.

This example illustrates the structural origin of the binomial coefficient. The combinatorial factor is not an auxiliary correction; it encodes the multiplicity of distinct histories compatible with the data. After normalization across admissible hypotheses, the common multiplicative factor cancels. Yet its role in the formation of posterior support remains conceptually significant: the discrete posterior is constructed from a counting structure before it becomes a normalized probability distribution.

For larger *n*, the same principle applies: posterior mass reflects both probabilistic weighting and combinatorial multiplicity. The minimal case n=3 makes this structural origin transparent without asymptotic smoothing.

## 3. Continuous Posterior and Structural Suppression

In the continuous formulation introduced by Laplace [1], the parameter *p* is treated as a continuous variable on the interval [0,1]. Under a uniform prior, the posterior density in the binomial setting is proportional to

(5) $$f\left(p|k,n\right)\propto p^{k}{\left(1-p\right)}^{n-k},$$

which, after normalization, yields a Beta distribution with parameters k+1 and n−k+1 [2]. This representation has become the standard analytic form of Bayesian updating in the binomial model.

From a computational and asymptotic perspective, the continuous posterior offers substantial advantages. It enables closed-form expressions for moments, supports smooth optimization procedures, and fits naturally into the general theory of parametric models. In this regime, posterior inference is expressed entirely through properties of a continuous density: its mode, mean, variance, and curvature.

However, the analytic representation also alters the representational structure of inference. In the discrete formulation, posterior weight arises from the aggregation of admissible outcome sequences. Each hypothesis corresponds to a finite collection of histories consistent with observed data. In the continuous formulation, by contrast, this combinatorial layer is no longer explicitly represented. The posterior is described solely through functional dependence on the parameter *p*, and multiplicities of admissible histories are absorbed into analytic normalization.

This transition can be understood as a form of structural suppression. The binomial coefficient, which counts admissible histories, disappears after normalization, and posterior mass is distributed according to the smooth curvature of the likelihood function. While this produces a mathematically elegant representation, it no longer distinguishes between individual admissible configurations at the representational level.

The passage from summation to integration can be written formally as replacing a finite sum by an integral:

(6) $$\sum_{i=0}^{N}p_{i}^{k}{\left(1-p_{i}\right)}^{n-k}\;⟶\;\int_{0}^{1}p^{k}{\left(1-p\right)}^{n-k}\;dp.$$

This change presupposes a shift from counting over finitely many admissible hypotheses to integration with respect to a continuous measure. In the limit of infinite resolution, discrete weights approximate a continuous density, yet at any finite resolution the two constructions remain distinct.

For large sample sizes, the continuous posterior admits a local quadratic approximation around its maximizer. Writing the log-posterior (up to an additive constant) as

(7) ℓ(p)=klogp+(n−k)log(1−p),

a second-order expansion around the maximizer yields a Gaussian approximation with variance of order O(1/n). In this analytic regime, posterior behavior is governed by curvature properties rather than combinatorial multiplicities. The representation of uncertainty becomes local and differential rather than discrete and enumerative. The implications of this representational shift under finite conditions are examined in the subsequent sections.

## 4. Numerical Regimes Under Finite Resolution

To make the structural distinction between discrete and continuous representations explicit, it is useful to examine several numerical regimes in which data size and grid resolution interact.

### 4.1. Moderate Sample Size with Increasing Resolution

Let n=20 and k=7. The continuous posterior density is proportional to $p^{7}{\left(1-p\right)}^{13}$, with mean

$$E\left[p\right]=\frac{k+1}{n+2}=\frac{8}{22}\approx 0.364.$$

Consider discrete grids with resolutions N=20, N=50, and N=200.

For N=20, admissible parameter values are spaced by 0.05. The discrete posterior mean differs noticeably from the continuous mean, and posterior mass is distributed over a relatively small number of admissible hypotheses.

For N=50, the spacing decreases to 0.02. The discrete posterior becomes closer to the continuous density in both mean and overall shape, though minor deviations remain due to finite alignment.

For N=200, the discrete posterior approximates the continuous density closely. At this resolution, differences are primarily quantitative rather than qualitative.

This regime illustrates standard convergence under refinement. However, convergence does not guarantee structural equivalence at finite resolution.

### 4.2. Increasing Sample Size at Fixed Resolution

Now fix the resolution at N=20 and increase sample size. Let n=100 and k=35. The continuous posterior becomes more sharply concentrated near p≈0.35. Because grid spacing remains 0.05, the posterior peak may lie between admissible grid points. The discrete posterior must allocate mass to the nearest admissible hypotheses. As sample size increases further, the continuous posterior sharpens, while the discrete posterior remains constrained by fixed grid spacing.

This produces a regime in which posterior concentration increases but resolution does not. In this setting, the discrete posterior no longer behaves as a smooth approximation of the continuous density; instead, it exhibits stepwise shifts as the most likely admissible hypothesis changes.

### 4.3. Sharp Posterior Regime

Consider n=100 and k=5. The continuous posterior is sharply concentrated near p=0.05. For coarse resolution such as N=10, admissible values are 0.0,0.1,0.2,…. The discrete posterior must assign maximal weight to either 0.0 or 0.1. In this case, representational constraints dominate posterior behavior.

If resolution is increased while sample size remains fixed, the discrete posterior progressively aligns with the continuous density. But when resolution is held fixed and sample size increases, divergence can become more pronounced.

### 4.4. Regime Interaction Between Sample Size and Resolution

The preceding examples suggest that posterior behavior under finite resolution depends on the joint scaling of sample size *n* and grid resolution *N*. Three scaling patterns can be distinguished.

First, resolution may increase faster than sample size. In this configuration, grid spacing decreases sufficiently rapidly so that the condition $\Delta p\ll \sigma_{p}$ is maintained as *n* grows. The discrete posterior then tracks the continuous density closely and divergence effects diminish with increasing data.

Second, sample size may increase while resolution remains fixed. In this case, posterior concentration increases while grid spacing remains constant. The inequality may reverse from $\Delta p\ll \sigma_{p}$ to $\Delta p\gg \sigma_{p}$, producing a qualitative shift from analytic-like behavior to structure-dominated behavior.

Third, both quantities may increase at comparable rates. In such situations, the ratio between posterior width and grid spacing may remain near unity over a range of values. This intermediate scaling generates a persistent mesoscopic regime in which structural and analytic components remain simultaneously active.

These scaling patterns show that divergence is not merely a static property of a fixed grid, but a dynamic effect determined by how representational and inferential scales evolve together.

### 4.5. Posterior Width as a Structural Indicator

The notion of posterior width provides a convenient structural indicator for distinguishing regimes. In the binomial setting, posterior width decreases as evidence accumulates. When grid spacing remains larger than this intrinsic scale, structural admissibility dominates. When grid spacing falls below this scale, analytic curvature governs posterior behavior. Thus, the relation between posterior width and representational resolution provides a quantitative criterion for regime classification and prepares the ground for the explicit analysis of divergence in the next section.

## 5. Divergence and the Mesoscopic Regime

The purpose of this section is to make explicit that divergence between discrete and continuous posteriors is a structural phenomenon rather than a numerical artifact. The present analysis is not intended to describe a specific computational methodology, but to examine the structural implications of finite representations independently of particular algorithmic implementations. The numerical regimes described above suggest that divergence between discrete and continuous posteriors is not a binary phenomenon but depends on the relative scale of two quantities: the concentration of the posterior distribution and the spacing of the parameter grid.

To formalize this intuition, consider the binomial posterior density under a continuous formulation. In more general settings with infinite parameter spaces, any practical representation necessarily involves a finite approximation, for example through truncation or discretization, which reduces the problem to a finite representational scheme.For large *n*, the Beta posterior is approximately normal with variance of order

(8) $$\mathrm{Var}\left(p|k,n\right)∼\frac{p(1-p)}{n},$$

so that the characteristic width scales as

(9) $$\sigma_{p}∼O\left(\frac{1}{\sqrt{n}}\right).$$

In contrast, the discrete representation imposes a fixed resolution

(10) $$\Delta p=\frac{1}{N}.$$

The relative magnitude of these two scales determines the regime of inference. When $\Delta p\ll \sigma_{p}$, multiple admissible hypotheses fall within the region of high posterior density and the discrete posterior behaves as a smooth approximation to the continuous density. When $\Delta p\gg \sigma_{p}$, posterior mass concentrates on a small number of admissible grid points and structural constraints dominate. The intermediate case $\Delta p∼\sigma_{p}$ defines a mesoscopic regime in which representational resolution and posterior concentration are comparable.

This divergence is not caused by inconsistency in Bayesian updating. Rather, it arises from the interaction between finite representational resolution and posterior concentration. The continuous regime assumes infinite divisibility of the parameter space; the discrete regime enforces admissible hypotheses at fixed spacing. When the intrinsic scale of the posterior matches the imposed representational scale, neither description fully absorbs the other.

*Order of Limits and Regime Pathways*

The regime classification depends not only on relative magnitude at a fixed point, but also on the order in which limits are taken. If resolution increases first, that is, if N→∞ while *n* remains fixed, the discrete posterior approaches the continuous density and structural distinctions become progressively negligible. If sample size increases first while resolution remains fixed, posterior concentration intensifies without corresponding refinement, and structural constraints become dominant. If *n* and *N* increase simultaneously, different scaling relations may preserve or eliminate the mesoscopic regime; for example, if $N∼\sqrt{n}$, grid spacing and posterior width remain comparable and mesoscopic behavior persists across increasing data volumes. These distinct limit pathways show that asymptotic equivalence does not uniquely determine finite-scale behavior.

*Structural and Representational Contributions to Divergence*

The divergence between discrete and continuous posteriors under finite conditions reflects the interaction of two distinct components of inference. One component is associated with the amount of information contained in the observed data. As more observations are accumulated, posterior distributions become increasingly concentrated. This concentration reflects genuine informational refinement. The other component is associated with the structure of the parameter representation itself. When the parameter space is discretized, admissible hypotheses are separated by a fixed spacing. This spacing introduces a structural constraint that may limit how finely posterior concentration can be expressed.

When posterior concentration remains broader than the representational spacing, discrete and continuous descriptions behave similarly. When posterior concentration becomes sharper than the available resolution, structural constraints determine how posterior mass is allocated. In intermediate configurations, both effects are simultaneously active.

In this perspective, divergence does not arise from inconsistency of Bayesian updating, but from the interaction between informational refinement and representational limitation. Even as data sharpen posterior beliefs, the structure of admissible hypotheses may shape how that sharpening is expressed.

Importantly, the transition between regimes is gradual rather than abrupt. As *n* increases while *N* remains fixed, posterior width decreases and may eventually fall below grid spacing, producing a shift from analytic-dominated behavior to structure-dominated behavior. Conversely, increasing *N* at fixed *n* moves the system toward the continuous regime. In this sense, divergence between discrete and continuous posteriors may reflect representational differences that are not captured by asymptotic convergence arguments alone.

## 6. The Scale-Dependent Role of the Prior: From Structure to Form

The regime analysis developed in the previous section was formulated in terms of the relation between posterior concentration and representational resolution. Posterior concentration, however, is not determined solely by sample size. In the binomial setting with a Beta prior, it also depends on prior strength, introducing a second structural scale into Bayesian inference.

Let the prior be given by a Beta distribution with parameters α and β. These parameters admit the standard interpretation as effective pseudo-counts representing accumulated prior contributions [2]. Denote total prior strength by

S=α+β.

Under Bayesian updating, posterior parameters become

$$\alpha^{\prime }=\alpha +k,\;\beta^{\prime }=\beta +\left(n-k\right).$$

The relative magnitude of *S* and *n* determines how strongly the prior influences posterior concentration and shape.

When prior strength is small relative to sample size, the posterior is primarily shaped by data, and structural distinctions induced by finite resolution may become visible. When prior strength dominates sample information, analytic smoothing induced by the prior suppresses structural variation even if grid resolution is moderate.

An intermediate case arises when prior strength and sample size are comparable. In this configuration, posterior behavior depends simultaneously on prior influence and representational resolution. Structural and analytic components interact, producing regime-dependent behavior that cannot be attributed to resolution or prior alone.

Combining the resolution scale discussed previously with prior strength yields a two-dimensional regime structure. Extension to multidimensional parameter spaces introduces additional geometric and representational considerations, and is beyond the scope of the present analysis, which focuses on the one-dimensional case in order to isolate the structural effect.Posterior behavior is determined jointly by representational resolution, sample size, and prior magnitude. Different combinations of these quantities produce structure-dominated, form-dominated, or mesoscopic regimes. Divergence between discrete and continuous posteriors under finite conditions therefore reflects not only grid effects but also the scale at which prior information interacts with data.

In this sense, the prior functions as a scale-dependent modifier of Bayesian inference. It influences not merely posterior location but the regime in which inference operates.

*Posterior Concentration Under Prior Influence*

The influence of prior strength on posterior concentration can be made more explicit. In the binomial model with a Beta prior, the posterior variance is

$$\mathrm{Var}\left(p|k,n\right)=\frac{\left(\alpha +k)(\beta +n-k\right)}{{\left(\alpha +\beta +n\right)}^{2}\left(\alpha +\beta +n+1\right)}.$$

Let S=α+β denote total prior strength. For moderate or large values of *n* and/or *S*, the posterior variance scales approximately as

$$\mathrm{Var}\left(p|k,n\right)∼O\;\left(\frac{1}{n+S}\right),$$

so that the characteristic posterior width behaves as

$$\sigma_{p}\left(n,S\right)∼O\;\left(\frac{1}{\sqrt{n+S}}\right).$$

Thus, posterior concentration depends on the combined scale n+S, rather than on sample size alone. When S≪n, concentration is governed primarily by data; when S≫n, prior magnitude controls posterior width even as additional data are observed. This refinement modifies the regime comparison introduced earlier: the relevant concentration scale is not simply $1/\sqrt{n}$, but $1/\sqrt{n+S}$.

## 7. Conclusions

The central result of this work is the identification of a mesoscopic regime of Bayesian inference, in which representational resolution and posterior concentration become comparable, and neither discrete nor continuous descriptions alone provide a complete account. This result shows that Bayesian updating, even in the classical binomial setting, admits distinct representational regimes that differ structurally despite sharing the same likelihood. By distinguishing between discrete and continuous formulations, the analysis reveals how posterior behavior depends on the interaction between resolution, sample size, and prior strength. Extensions to more general models and broader applications are beyond the scope of the present study and are left for future work. Accordingly, the present work should be interpreted as a conceptual analysis rather than as a prescription for a general computational procedure, with the aim of identifying structural features of Bayesian inference under finite representations.

The study demonstrates that qualitative divergence between discrete and continuous posteriors may arise under finite conditions. Such divergence is not a consequence of inconsistent updating, but of the interaction between posterior concentration and representational resolution. When grid spacing is large relative to posterior width, structural constraints dominate; when resolution is sufficiently fine, analytic smoothing prevails; and when these scales are comparable, an intermediate mesoscopic regime emerges.

The analysis further shows that posterior behavior depends not only on resolution and sample size but also on prior strength. The prior introduces an additional structural scale that influences whether inference operates in a structure-dominated, form-dominated, or intermediate regime. Resolution, data volume, and prior magnitude jointly define a two-dimensional scale structure governing Bayesian inference under finite conditions.

In addition to clarifying the interaction between resolution, sample size, and prior strength, the identification of a mesoscopic regime suggests that finite-scale Bayesian inference may exhibit structural regularities that remain invisible in purely asymptotic analyses. When structural and analytic components are simultaneously active, intermediate regimes can give rise to stable patterns of behavior that do not reduce to either limiting description. Exploring such regime-dependent structures provides a principled motivation for studying finite-resolution inference beyond questions of numerical approximation alone.

## Data Availability

The original contributions presented in this study are included in the article. Further inquiries can be directed to the corresponding author.
